# Antibacterial Effect of Pomegranate Juice on *Listeria innocua* and *E. coli* in Different Media

**DOI:** 10.3390/foods12173247

**Published:** 2023-08-29

**Authors:** Zhaojun Ban, Lihua Fan, Jun Song, Sherry Fillmore, Junfeng Guan

**Affiliations:** 1 School of Biological and Chemical Engineering, Zhejiang University of Science and Technology, Hangzhou 310023, China; bzhaojun@163.com; 2 Kentville Research and Development Centre, Agriculture and Agri-Food Canada, 32 Main Street, Kentville, NS B4N 1J5, Canada; jun.song@agr.gc.ca (J.S.); sherry.fillmore@agr.gc.ca (S.F.); 3 Hebei Academy of Agriculture and Forestry Sciences, Shijiazhuang 050050, China; junfeng-guan@263.net

**Keywords:** pomegranate juice, natural antimicrobials, *Listeria innocua*, *Escherichia coli*

## Abstract

The antibacterial effect of pomegranate juice (PJ) at six concentrations (0, 10, 20, 30, 40, and 50%) against *Listeria innocua* and *Escherichia coli* was investigated in distilled water (DW) and bacterial culture broth. *L. innocua* and *E. coli* at approximately 10^5^ cfu mL^−1^ were inoculated in PJ samples and incubated at 4, 25, and 37 °C for 0, 6, 24, and 48 h. The bacterial population and pH of culture media were measured at each removal. Results indicated that the antibacterial effect of PJ was dependent upon bacteria species, juice concentration, incubation temperature, and growth medium. Higher juice concentration and incubation temperature resulted in increased antibacterial effects. Bacterial populations were decreased more significantly in DW systems than in the culture broth, while *L. innocua* was more sensitive to PJ than *E. coli* in the DW systems. Regardless of PJ concentrations in DW systems, *L. innocua,* initially inoculated at approximately 10^5^ cfu mL^−1^, was reduced to undetectable levels at 25 and 37 °C within 24 h. The growth of *L. innocua* and *E. coli* was significantly inhibited in bacterial culture broth containing ≥ 20% PJ (*p* < 0.001). This study provides insight into the potential application of PJ in food and beverage products for food protection.

## 1. Introduction

The fruit of pomegranate (*Punica granatum*) comprises peel containing polyphenols, minerals, and complex polysaccharides. The edible portion of pomegranates contains organic acids, sugars, vitamins, polysaccharides, polyphenols, and minerals, although the chemical composition of the fruit varies depending on the cultivar, environmental conditions, maturity, and postharvest treatments [1,2,3]. Pomegranates have become a valuable fruit because of the potential health-promoting benefits of their bioactive compounds, including tannins, flavonoids, alkaloids, organic acids, triterpenes, and steroids in different parts of the fruit. Pomegranate fruits are moderate in taste, with sour notes from the acidic ellagitannins contained in the juice. The most abundant phytochemicals in pomegranate juice are polyphenols, including the hydrolyzable tannins called ellagitannins, formed when ellagic acid and gallic acid bind with a carbohydrate to form pomegranate ellagitannins, also known as punicalagins [3]. The red color of the juice is attributed to anthocyanins. In recent years, pomegranate juice (PJ) has gained popularity among consumers for its antioxidant, antimicrobial, anticancer, anti-inflammatory, and antiviral properties [3,4,5,6]. 

*Listeria monocytogenes* and *Escherichia coli* are regarded as significant causes of foodborne illness and have been a major concern for consumers and the food industry. *L. monocytogenes*, a Gram-positive pathogen, is able to grow at refrigerator temperatures, causing contamination for many fresh and processed food products [7]. This bacterium is responsible for the disease listeriosis, which has a high mortality rate [8]. *L. innocua*, a surrogate for *L. monocytogenes*, is non-hemolytic and non-pathogenic and can be found with *L. monocytogenes* in the same food or processing environment. Due to their high phenotypic similarity, *L. innocua* is often used as an indicator for *L. monocytogenes* [9,10]. *E. coli* O157:H7, a Gram-negative pathogen, is a foodborne pathogen exceptionally tolerant of acid pH and can survive at pH 3–4 [11,12]. *E. coli* can be found in a wide variety of foods and is regarded as an indicator of bacterial contaminants of fecal origin for quality assurance of municipal water and other food products [13,14]. 

Adding chemical preservatives to foods can reduce the risk of microbiological contamination. However, consumers have been demanding natural, healthy, and safe foods. The growing demand for developing natural antimicrobials is in response to the perceived risks associated with chemical residues in food [15,16,17]. Food product manufacturers are interested in utilizing natural plant-derived preservatives to inhibit bacteria such as *Listeria* spp. and *E. coli* as a means to reduce the use of synthetic additives and ensure food safety with extended shelf life [4,18]. Therefore, the objectives of this study were to investigate the antimicrobial effects of pomegranate juice at different concentrations against *L. innocua* and *E. coli* and determine the treatment effects in distilled water (DW) and bacterial culture broth systems at different temperatures.

## 2. Materials and Methods

### 2.1. Pomegranate Juice

Commercially processed 100% PJ (not from concentrate) without any food additives or preservatives was obtained from a local grocery supplier (Atlantic Superstore, New Minas, NS, Canada) and stored at 4 °C until use. Soluble solid content of PJ was measured using a digital hand-held refractometer (Model “Pocket PAL-1”, Atago^®^ Co., Inc., Tokyo, Japan), where approximately 0.3 mL of juice was placed onto the prism surface of the refractometer. Titratable acidity (TA) of PJ was measured with 0.5 N NaOH using the Metrohm Model 848 Titrino Plus (Herisau, Switzerland) expressed in citric acid equivalents TA (%). The pH of PJ was measured using a pH meter (Accumet Basic AB15, Fisher Scientific, Hampton, NH, USA). Total phenolic content (gallic acid equivalent) was determined using Folin-Ciocalteu Reagent, and gallic acid was used as standard [19].

### 2.2. Preparation of Bacterial Strains

*L. innocua* (ATCC33090) and *E. coli* (ATCC10798) were stored at −86 °C in Brain Heart Infusion broth (BHI) (Becton Dickinson and Company, Sparks, MD, USA) or Tryptone Soya Broth (TSB) (Oxoid Ltd., Hampshire, UK) containing 15% glycerol, respectively until use. Prior to the experiment, the working cultures were prepared by sub-culturing 100 µL of *L. innocua* stock culture in 10 mL BHI and 100 µL *E. coli* in 10 mL TSB, then incubated at 37 °C for 24 h. The working cultures were centrifuged at 11,500× *g* for 10 min, supernatants were removed, and bacterial cells were washed with sterile water then centrifuged again. The resulting pellets were made up to a 5 mL volume with sterile water and used for *L. innocua* or *E. coli* inoculum. Each inoculum was serially diluted and spiral plated in duplicate on BHI or TSB agar using a WASP 2 (Don Whitley Scientific Ltd., Bingley, UK) spiral plater and incubated at 37 °C for 48 h for enumeration of total population counts of inoculum. For each repeat, the new working cultures were prepared as described above to keep consistent bacterial populations of initial inoculum. 

### 2.3. Antibacterial Activity 

The antibacterial activity of six concentrations of PJ was investigated in bacterial culture broth and sterilized distilled water (DW) systems. At each concentration level, an appropriate volume of juice was added to the culture broth or DW in a sterile test tube to a final volume of 10 mL. The final concentrations of PJ were 0, 10, 20, 30, 40, and 50% (*v*/*v*). Each tube was then inoculated to obtain an initial inoculum level of approximately 10^5^ cfu mL^−1^. The samples were shaken well and incubated at 4, 25, or 37 °C to represent cold storage temperature, room temperature, and optimum bacterial growth temperature, respectively. The bacterial population was measured at 0, 6, 24, and 48 h following inoculation. At each sampling time, an aliquot of each sample was serially diluted with 0.1% sterile peptone water (PW, Difco, Becton Dickinson and Company, MD, USA), and the appropriate dilution was spiral plated onto the BHI or TSB agar using the 100 µL mode of the spiral plate system, WASP2 for enumeration of *L. innocua* and *E. coli,* respectively. After incubation at 37 °C for 24 h, visible colonies of *L. innocua* and *E. coli* on the surface of the BHI or TSB agar plates were counted using the aCOLyte colony counter (Synbiosis, Cambridge, UK). 

### 2.4. Statistical Analysis

The experimental design was a completely randomized 2 × 2 × 6 × 3 × 4 factorial with two bacteria species (*L. innocua* and *E. coli*), two culture media (bacteria culture broth and DW), six juice concentrations (0, 10, 20, 30, 40, and 50%), three incubation temperatures (4, 25, and 37 °C), and four removal times (0, 6, 24, and 48 h). Bacterial populations in this study were expressed as log cfu mL^−1^. Data were analyzed using the ANOVA directive and standard errors of mean (SEM) option of GenStat^®^ (12th Edition, VSN International Ltd., Hemel Hempstead, UK). All experiments were replicated three times.

## 3. Results 

### 3.1. Basic Chemical Contents of Pomegranate Juice

The chemical properties of 100% PJ used in our study were measured. The soluble solid content was 16 Brix (%), titratable acidity (TA) was 0.91% ± 0.10, pH value was 3.32 ± 0.07, and total phenolic content (gallic acid equivalent) was 1717.04 mg GAE l^−1^.

### 3.2. Antibacterial Effect of PJ against L. innocua in Brain Heart Infusion (BHI) Broth System

**Figure 1:**
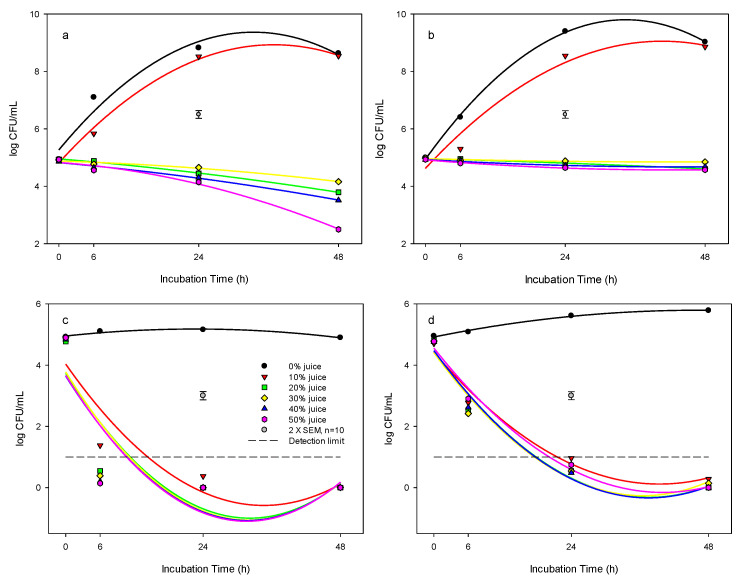
Antibacterial effect of pomegranate juice against *L. innocua* in: (**a**) BHI system at 37 °C, (**b**) BHI system at 25 °C, (**c**) DW system at 37 °C, and (**d**) DW system at 25 °C. The detection limit for *L. innocua* was <1 log cfu mL^−1^. Pomegranate juice concentrations are shown: (●) 0%, (▼) 10%, (■) 20%, (♦) 30%, (▲) 40%, (⬢) 50%. The vertical bars represent the standard errors of mean (SEM).

At 37 °C incubation temperature, PJ concentrations at 20, 30, 40, and 50% (*v*/*v*) in the BHI broth had a significant inhibitory effect on the growth of *L. innocua* (Figure 1a), where the bacterial populations were at 3.8, 4.1, 3.5, 2.5 logs, respectively, compared to the control of 8.6 log cfu mL^−1^ over the 48 h incubation. However, with PJ concentrations of 10% in the BHI broth, the growth of *L. innocua* followed the typical bacterial growth curve and reached 8.5 log cfu mL^−1^ after 24 h incubation, even though the bacterial population in the 10% PJ was slightly lower than that of the control at 6 and 24 h incubation. 

At 25 °C incubation temperature, the bacterial growth curves were similar to that of 37 °C for the PJ concentrations of 0 and 10%, with the population in the 10% suspension being lower than that of the 0% (control) at 6 and 24 h incubation (Figure 1b). Again, for treatments with PJ concentrations of 20, 30, 40, and 50%, the *L. innocua* counts were 4.6, 4.8, 4.7, and 4.6 logs, respectively, compared to the control of 9.03 log cfu mL^−1^ following 48 h at 25 °C, suggesting the strong inhibitory effects of PJ on *L. innocua*.

At 4 °C incubation temperature, there was no change in the population of *L. innocua* in 0, 10, and 20% PJ over time (Table 1). There was a slight decrease in the population in the 30, 40, and 50% PJ concentrations compared with control during the 48 h incubation. 

### 3.3. Antibacterial Effect of PJ against L. innocua in DW System

The response of *L. innocua* to the concentrations of PJ suspended in distilled water was different from that of PJ added in BHI. At 25 and 37 °C incubation temperatures, the populations of *L. innocua* were decreased to undetectable levels (<10 cfu mL^−1^) within 24 h at PJ concentrations ≥ 10% in distilled water (Figure 1c,d), suggesting an effective killing effect of PJ on *L. innocua*. At 4 °C, the population of *L. innocua* was also decreased by up to 1.5 or 3.3 logs following 6 or 24 h treatment, respectively, and was further reduced to undetectable levels within 48 h by PJ concentrations tested (Table 1). 

### 3.4. Antibacterial Effect of PJ against E. coli in Tryptone Soya Broth (TSB) System

**Figure 2:**
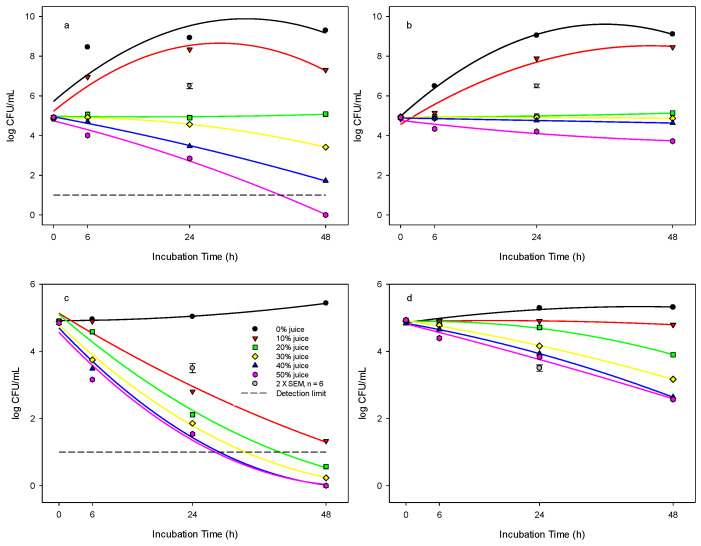
Antibacterial effect of pomegranate juice against *E. coli* in: (**a**) TSB system at 37 °C, (**b**) TSB system at 25 °C, (**c**) DW system at 37 °C, and (**d**) DW system at 25 °C. The detection limit for *E. coli* was <1 log cfu mL^−1^. Pomegranate juice concentrations are shown: (●) 0%, (▼) 10%, (■) 20%, (♦) 30%, (▲) 40%, (⬢) 50%. The vertical bars represent the standard errors of mean (SEM).

At 37 °C incubation temperature, the concentrations of PJ at 20, 30, 40, and 50% in the TSB suspension had a significant inhibitory effect on the growth of *E. coli* (Figure 2a). *E. coli* was counted at 5.0, 3.4, 1.7, and <1 log cfu mL^−1^, respectively, in comparison to 9.3 log cfu mL^−1^ in the control following 48 h incubation duration. In the control and 10% PJ concentration, the growth of *E. coli* followed the typical bacterial growth curve over the 48 h incubation, but the population in the 10% suspension remained lower than that of the control after incubation. We found that for the treatment with 20% PJ in TSB, the *E. coli* population did not change over time compared with the initial population inoculated at 5 log cfu mL^−1^, indicating an inhibitory effect rather than killing the bacteria. The killing effect was shown at PJ concentrations of 30, 40, and 50%, and the *E. coli* population was reduced significantly (*p* < 0.001) over time. The 50% PJ concentration resulted in the greatest reduction of *E. coli* to an undetectable level after 48 h incubation.

At 25 °C incubation temperature, the change in the *E. coli* population was similar to that of 37 °C for the concentrations of 0 and 10% (Figure 2b). Overall, for treatments with PJ concentrations of 20, 30, 40, and 50%, the *E. coli* growth was inhibited, and bacterial counts were determined at 5.1, 4.8, 4.6, and 3.7 logs, respectively, compared to 9.1 log cfu mL^−1^ in the control after 48 h incubation. It was found that PJ concentrations of 20, 30, and 40% had similar inhibitory effects on the *E. coli* population over time. 

At 4 °C incubation temperature, there was no change in the population of *E. coli* in the 0, 10, 20, 30, and 40% concentrations over time (Table 1). Compared to the control, only a 0.3 log cfu mL^−1^ decrease was found in the *E. coli* population following treatment with 50% PJ for 48 h. 

### 3.5. Antibacterial Effect of PJ against E. coli in DW System

The population of *E. coli* responded differently in the distilled water system compared to that in TSB. At 37 °C incubation temperature, there was a slight change in the population of *E. coli* inoculated in the 0% juice (control) over 48 h (Figure 2c). However, after PJ juice ≥ 10% was added to the DW system, the *E. coli* population significantly decreased over time compared with the control (*p* < 0.001). Furthermore, *E. coli* populations decreased to undetectable levels (<10 cfu mL^−1^) after being treated with PJ at concentrations ≥ 20% for 48 h.

At 25 °C incubation temperature, there were only slight changes in the population of *E. coli* inoculated in the control or 10% juice suspension over 48 h in distilled water (Figure 2d). The *E. coli* population decreased over time at the 20, 30, 40, and 50% concentrations, but not to the same degree as that for the 37 °C incubation. After 48 h incubation, the population of *E. coli* remained at 3.8, 3.1, 2.6, and 2.5 logs in samples treated with 20, 30, 40, and 50% PJ, respectively, compared to 5.3 log cfu mL^−1^ in the control. At 4 °C incubation temperature, there was only a slight change in the bacterial population in the 0, 10, 20, and 30% PJ concentrations over time (Table 1). It was found that the initial *E. coli* population at 4.9 logs was decreased to 4.1 or 3.7 log cfu mL^−1^ over 48 h incubation in the 40 and 50% PJ, respectively. 

### 3.6. Lower pH and Higher Concentration of PJ Contribute to Antimicrobial Effect

**Figure 3:**
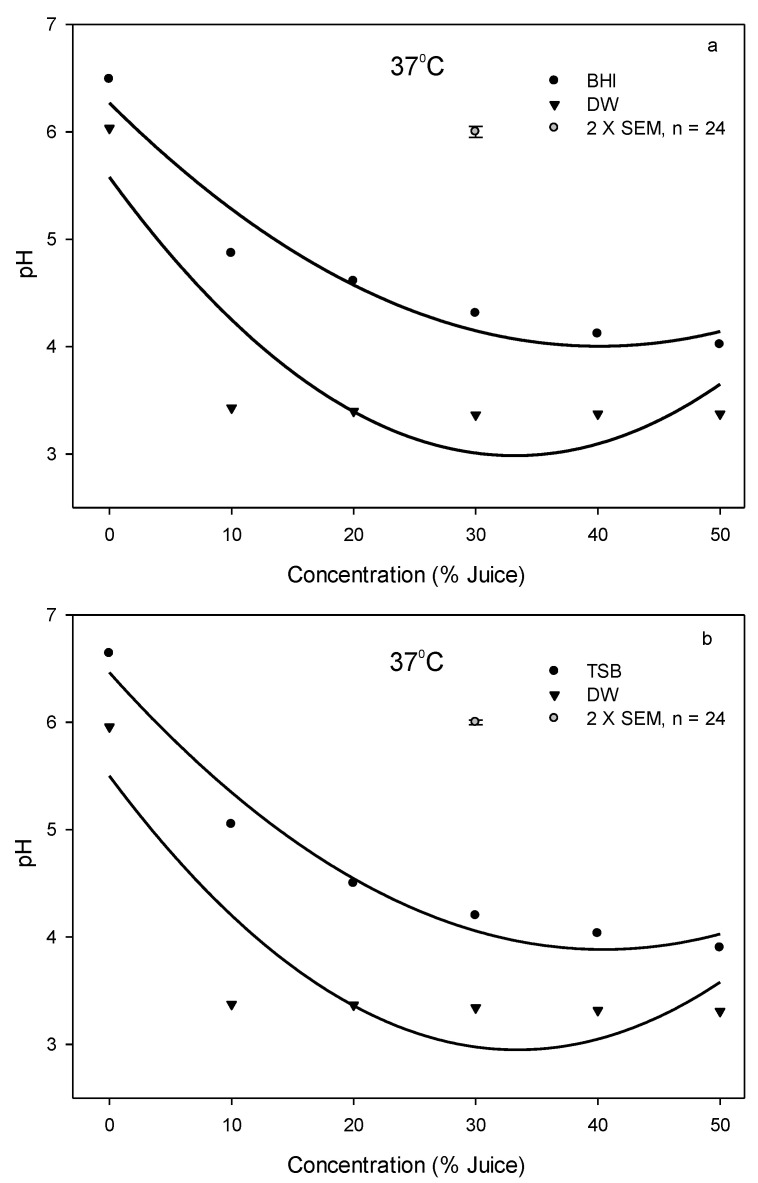
Changes of pH value with an addition of pomegranate juice in the bacterial culture broth or DW system at 37 °C, (**a**) (●) BHI or (▼) DW used for *L. innocua*, (**b**) (●) TSB or (▼) DW used for *E. coli*. The vertical bars represent the standard errors of mean (SEM).

**Figure 4:**
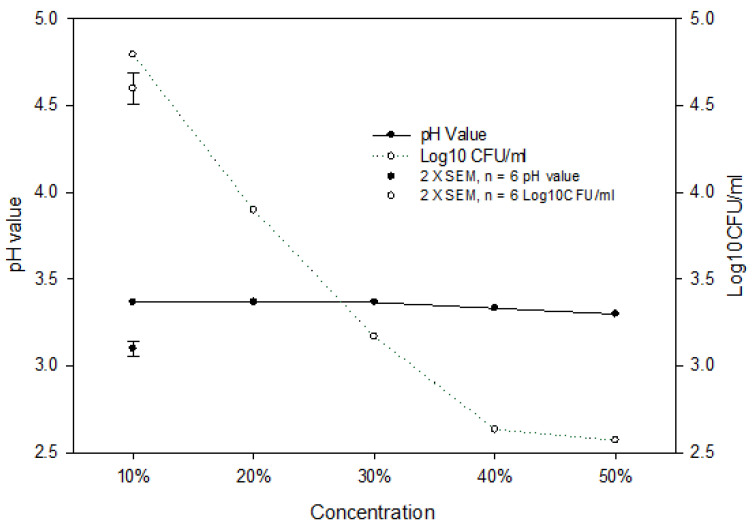
pH change and antimicrobial effect of pomegranate juice at different concentrations on *E. coli* population in DW system at 25 °C for 48 h. (●) pH values, (○) *E. coli* population. The vertical bars represent the standard errors of mean (SEM).

Both bacteria were more effectively inhibited and/or killed in DW systems than in culture broth systems because of a lack of nutrients in DW to support bacterial growth, and the pH of DW systems was lowered by the addition of PJ to a greater degree ranging from 3.3 to 3.4 than that in culture broth systems ranging from 4–5 at 37 °C (Figure 3). The change of pH value due to the addition of PJ at 4 or 25 °C in DW and culture broth system was similar to that at 37 °C. In addition, we investigated the pH change and PJ concentration on *E. coli* growth in DW at 25 °C for 48 h (Figure 4) and found that pH values remained at 3.3–3.4 regardless of PJ concentration levels of 10, 20, 30, 40, and 50%. However, the increase in juice concentration resulted in a decrease in the *E. coli* population following the 48 h incubation, suggesting that while pH was one of the factors contributing to the antimicrobial effect, other chemical components in PJ also played an important role in inhibiting and/or killing bacteria. 

## 4. Discussion

Pomegranate fruits are known as a good source of healthy antioxidants, including polyphenols and anthocyanin [3,20]. While these active compounds are becoming increasingly popular for their health benefits, some of them also demonstrate antimicrobial properties for food preservation. Pomegranate and pomegranate extract can be in the form of liquid or powder; these natural ingredients extend the shelf life of food and beverage products without adverse effects on taste and visuals while, at the same time, allowing for convenience and safety [21,22]. Saeed et al. [18] reported that pomegranate peel extract was an effective antimicrobial coating material for tomatoes. They found that the coated tomatoes were firmer and had more lycopene content than the control, and the coating materials did not affect the sensory profile of tomatoes. In this study, the antibacterial potential of PJ at different concentrations against *L. innocua* and *E. coli* in both water and culture broth systems (to imitate food matrix) at 4, 25, and 37 °C (to reflect cold storage temperature, room temperature, and suitable temperature for growth of some bacteria, respectively) were investigated, which were different from the research reported previously. PJ showed antibacterial effects against Gram-positive *L. innocua* and Gram-negative *E. coli*, while both bacteria were inhibited and killed more effectively in DW systems than in culture broth systems. PJ has intrinsic characteristics with low pH values and high titratable acidity that create a hostile environment for bacterial growth and survival. Microbial response to acidic pH is a major factor controlling the growth and survival of bacteria in foods and acidic body environments [23]. *L. innocua* was found to be more sensitive to PJ than *E. coli,* as significant *L. innocua* reductions (*p* < 0.001) were shown following the additions of PJ for 6 h at three temperatures tested in DW systems. Gahan et al. [24] reported that the lethal acid stress for *L. monocytogenes* is at pH 3.5 without the treatment of adaptive acid tolerance response by using nutrient broth systems. The *L. innocua* did not tolerate the acidified conditions with limited nutrients. *E. coli* could better tolerate the lower pH in comparison to *L. innocua*. Miller and Kaspar [25] reported that *E. coli* O157:H7 could be detected in apple cider with a pH of 3.6 stored at 8 °C and was shown not to be destroyed within the shelf life of apple cider at pH 2 to 4. Nikaido [26] reported that the resistance mechanisms of Gram-negative bacteria to lower pH are more complicated than Gram-positive bacteria due to the different characteristics of their cell wall composition. As reported by Seltmann and Holst [27], the cell wall of Gram-positive bacteria has much more peptidoglycan than that of Gram-negative bacterial cells, providing increased rigidity. The difference in cell wall structures between *Listeria* spp. and *E. coli* may lead to different effects when the bacteria are subjected to antimicrobial compounds [28]. 

It is worth noting that the higher concentration of PJ showed greater antibacterial effects than the lower concentration of PJ at the same pH level (Figure 4). Bacterial reductions were measured at the higher PJ concentrations, implying that certain chemical compounds, such as phenolics in the PJ (measured at 1717.04 mg GAE L^−1^ in this study), also contribute to the antibacterial actions. The primary polyphenols of 4-Hydroxy-3-Methoxybenzoate, epicatechin, catechin, rutin, ferulic acid, P-coumaric acid, and cinnamic acid in pomegranate juice concentrate (PJC) were determined [29]. PJC demonstrated potential antibacterial effects on *Streptococcus mutans* and *Aeromonas hydrophila*. Wu et al. [30] studied the inhibitory activity of aqueous extracts of pomegranate peel products and juice powder against *Salmonella enterica*, indicating that the level of *Salmonella* population reduction correlated positively with the total polyphenolic content and titratable acidity in the treatment system. Phenolics with hydrophobicity could act efficiently at the bacterial membrane–water interface by embedding in the membrane, thereby impairing the cell membrane [31]. Apostolidis et al. [32] and Lin et al. [33] reported that phenolic phytochemicals and organic acids can cause hyper-acidification via proton donation at the plasma membrane interface of the microorganism and intracellular cytosolic acidification and exposure to low pH can cause sub-lethal injury to cell membranes, resulting in disruption of proton motive force owing to loss of H^+^-ATPase required for ATP synthesis. This damage may make the bacteria more susceptible to the phenolic compounds in the PJ. Türkyilmaz et al. [22] reported that the antimicrobial activities of PJ were due to tannins such as ellagitannins and other high molecular weight tannins. Reddy et al. [21] determined that gallagic acid and punicalagin could inhibit the growth of *E. coli*, *P. aeruginosa*, and *C. neoformans* with IC_50_ values lower than 15 µg mL^−1^. 

Different treatment temperatures had impacts on the antimicrobial activities of PJ against *L. innocua* and *E. coli*. Higher incubation temperatures resulted in stronger bactericidal effects. In this study, both bacteria were less affected by PJ at 4 °C. With abundant nutrients and optimum temperature, the newly formed cells of the bacteria are active and multiply quickly. The cell wall of newly formed cells is more easily destroyed than that of older cells [34]. Aronsson [35] reported that phospholipids in bacterial cell membranes are closely packed into a rigid structure, but at high temperatures, they are arranged in a less ordered manner. This could be the reason for the increased antibacterial action of PJ at high temperatures. In addition, at high temperatures, bacterial cell membranes may assist in transporting organic acids into the cells [36].

Our study demonstrated the antimicrobial effects of PJ against *L. innocua* and *E. coli* in bacterial culture broth systems, suggesting that PJ has antimicrobial potential in the food system. Xylia et al. [37] also reported that the washing treatments with PJ (1:10) resulted in a significant decrease of *E. coli* and *L. monocytogenes* population on shredded carrots, while total carotenoids, ascorbic acid, total soluble solids, and titratable acidity were not different after six days of storage of samples. Pomegranate has been used in the food industry, including juice for drinking and its extract for extending the shelf life of foods. PJ and its extracts present high antioxidant and antimicrobial activity, which make them excellent candidates as natural additives or active compounds in the food and food packaging industries [38]. To better understand the mode of action of the antimicrobial properties of pomegranates, further investigation on bioactive compounds in pomegranates and the correlation between phenolic compounds and antimicrobial activity are needed. In addition, the potential applications of pomegranate juice/extract in food systems warrant more research. 

## 5. Conclusions

The antimicrobial effects of PJ were dependent upon the juice concentration, culture conditions (temperature, matrix, and time), as well as bacterial species. Increased juice concentration and treatment temperature resulted in a greater antibacterial effect. *L. innocua* and *E. coli* were more effectively killed in the DW systems than in their culture broth, while *L. innocua* was more sensitive to PJ compared to *E. coli*. Low pH value and chemical compounds in PJ contributed to the antimicrobial effect. This study provides useful information for the food industry to develop pomegranate juice and/or its extracts as healthy food/beverage as well as natural food additives for food protection. 

## Figures and Tables

**Table 1:** Antibacterial effect of pomegranate juice against *L. innocua* and *E. coli* at 4 °C.

Medium	Concentration (%)	*L. innocua* Population (log cfu mL^−1^) ^a^				*E. coli* Population (log cfu mL^−1^) ^a^			
		0 h	6 h	24 h	48 h	0 h	6 h	24 h	48 h
BHI	0	4.96	4.93	4.92	4.90	4.88	4.89	4.87	4.86
TSB ^b^	10	4.95	4.95	4.92	4.92	4.89	4.85	4.95	4.89
	20	4.88	4.83	4.84	4.80	4.89	4.99	4.90	4.87
	30	4.95	4.85	4.71	4.53	4.86	4.94	4.96	4.88
	40	4.90	4.80	4.52	4.43	4.91	4.90	4.85	4.81
	50	4.93	4.71	4.46	4.29	4.91	4.66	4.64	4.50
DW	0	5.00	4.87	4.87	4.86	4.89	4.97	4.87	4.89
	10	4.98	3.33	1.57	- ^c^	4.91	4.89	4.83	4.81
	20	4.78	3.44	1.56	-	4.85	4.88	4.84	4.83
	30	4.80	3.50	2.18	-	4.85	4.87	4.68	4.67
	40	4.82	3.43	1.66	-	4.86	4.59	4.22	4.11
	50	4.88	3.50	1.76	-	4.88	4.19	3.90	3.76

^a^ The experiments were repeated three times, and the data were expressed as means. ^b^ BHI: bacterial culture broth used for *L. innocua*; TSB: bacterial culture broth used for *E. coli.* ^c^ below the detection limit (<1 log cfu mL^−1^).

## Data Availability

The datasets generated for this study are available on request to the corresponding author.

## References

[1] Christaki E.V., Bonos E.M., Florou-Paneri P.C. (2011). Dietary benefits of pomegranates in humans and animals. J. Food Agric. Environ..

[2] Schwartz E., Tzulker R., Glazer I., Bar-Ya’akov I., Wiesman Z., Tripler E., Bar-Ilan I., Fromm H., Borochov-Neori H., Holland D. (2009). Environmental conditions affect the color, taste, and antioxidant capacity of 11 pomegranate accessions’ fruits. J. Agric. Food Chem..

[3] Gómez Caravaca A.M., Verardo V., Toselli M., Segura Carretero A., Fernández Gutiérrez A., Caboni M.F. (2013). Determination of the major phenolic compounds in pomegranate juices by HPLC−DAD−ESI-MS. J. Agric. Food Chem..

[4] Lucas D.L., Were L.M. (2009). Anti-*listeria monocytogenes* activity of heat-treated lyophilized pomegranate juice in media and in ground top round beef. J. Food Prot..

[5] Shema-Didi L., Sela S., Ore L., Shapiro G., Geron R., Moshe G., Kristal B. (2012). One year of pomegranate juice intake decreases oxidative stress, inflammation, and incidence of infections in hemodialysis patients: A randomized placebo-controlled trial. Free Radic. Biol. Med..

[6] Viuda-Martos M., Fernández-López J., Pérez-Álvarez J.A. (2010). Pomegranate and its many functional components as related to human health: A review. Compr. Rev. Food Sci. Food Saf..

[7] Nuñez De Gonzalez M.T., Keeton J.T., Acuff G.R., Ringer L.J., Lucia L.M. (2004). Effectiveness of acidic calcium sulfate with propionic and lactic acid and lactates as postprocessing dipping solutions to control *Listeria monocytogenes* on frankfurters with or without potassium lactate and stored vacuum packaged at 4.5 °C. J. Food Prot..

[8] Vázquez-Boland J.A., Kuhn M., Berche P., Chakraborty T., Domínguez-Bernal G., Goebel W., González-Zorn B., Wehland J., Kreft J. (2001). *Listeria* pathogenesis and molecular virulence determinants. Clin. Microbiol. Rev..

[9] Gómez D., Ariño A., Carramiñana J.J., Rota C., Yangüela J. (2012). Sponge versus mini-roller for the surface microbiological control of *Listeria monocytogenes*, total aerobic mesophiles and Enterobacteriaceae in the meat industry. Food Control.

[10] Margolles A., Mayo B., De Los Reyes-Gavilán C.G. (2001). Susceptibility of *Listeria monocytogenes* and *Listeria innocua* strains isolated from short-ripened cheeses to some antibiotics and heavy metal salts. Food Microbiol..

[11] Conner D.E., Kotrola J.S. (1995). Growth and survival of *Escherichia coli* O157:H7 under acidic conditions. Appl. Environ. Microbiol..

[12] Gabriel A.A., Nakano H. (2010). Responses of *E. coli* O157:H7, *L. monocytogenes* 1/2 c and *Salmonella enteritidis* to pH, aw and temperature stress combinations. Food Control.

[13] Doyle M.P. (1991). *Escherichia coli* O157: H7 and its significance in foods. Int. J. Food Microbiol..

[14] Lang M.M., Harris L.J., Beuchat L.R. (2004). Survival and recovery of *Escherichia coli* O157:H7, *Salmonella*, and *Listeria monocytogenes* on lettuce and parsley as affected by method of inoculation, time between inoculation and analysis, and treatment with chlorinated water. J. Food Prot..

[15] Gould G.W. (1996). Industry perspectives on the use of natural antimicrobials and inhibitors for food applications. J. Food Prot..

[16] De La Torre Torres J., Gassara F.E., Kouassi A.P., Brar S.K., Belkacemi K. (2017). Spice use in food: Properties and benefits. Crit. Rev. Food Sci. Nutr..

[17] Su X., Howell A.B., D’Souza D.H. (2010). Antiviral effects of cranberry juice and cranberry proanthocyanidins on foodborne viral surrogates—A time dependence study in vitro. Food Microbiol..

[18] Saeed M., Azam M., Ahmad T., Akhtar S., Hussain M., Nasir S., Ain Q.U. (2022). Utilization of pomegranate peel extract as antifungal coating agent against *Fusarium oxysporum* on tomatoes. J. Food Process. Preserv..

[19] Kalt W., Howell A., Duy J.C., Forney C.F., McDonald J.E. (2001). Horticultural factors affecting antioxidant capacity of blueberries and other small fruit. HortTechnology.

[20] Kostka T., Ostberg-Potthoff J.J., Briviba K., Matsugo S., Winterhalter P., Esatbeyoglu T. (2020). Pomegranate (*Punica granatum* L.) extract and its anthocyanin and copigment fractions-free radical scavenging activity and influence on cellular oxidative stress. Foods.

[21] Reddy M.K., Gupta S.K., Jacob M.R., Khan S.I., Ferreira D. (2007). Antioxidant, antimalarial and antimicrobial activities of tannin-rich fractions, ellagitannins and phenolic acids from *Punica granatum* L. Planta Medica.

[22] Türkyilmaz M., Taǧi Ş., Dereli U., Özkan M. (2013). Effects of various pressing programs and yields on the antioxidant activity, antimicrobial activity, phenolic content and colour of pomegranate juices. Food Chem..

[23] Kroll R., Patchett R. (1992). Induced acid tolerance in *Listeria monocytogenes*. Lett. Appl. Microbiol..

[24] Gahan C., O’Driscoll B., Hill C. (1996). Acid adaptation of *Listeria monocytogenes* can enhance survival in acidic foods and during milk fermentation. Appl. Environ. Microbiol..

[25] Miller L.G., Kaspar C.W. (1994). *Escherichia coli* O157: H7 acid tolerance and survival in apple cider. J. Food Prot..

[26] Nikaido H. (2003). Molecular basis of bacterial outer membrane permeability revisited. Microbiol. Mol. Biol. Rev..

[27] Seltmann G., Holst O. (2002). The Bacterial Cell Wall.

[28] Puupponen-Pimiä R., Nohynek L., Meier C., Kähkönen M., Heinonen M., Hopia A., Oksman-Caldentey K.M. (2001). Antimicrobial properties of phenolic compounds from berries. J. Appl. Microbiol..

[29] Habib H.M., El-Gendi H., El-Fakharany E.M., El-Ziney M.G., El-Yazbi A.F., Al Meqbaali F.T., Ibrahim W.H. (2023). Antioxidant, Anti-Inflammatory, Antimicrobial, and Anticancer Activities of Pomegranate Juice Concentrate. Nutrients.

[30] Wu W., Mis Solval K., Chen J. (2022). Inhibitory activity of aqueous extracts of pomegranate peel products and juice powder against *Salmonella enterica*. LWT-Food Sci. Technol..

[31] Vattem D.A., Lin Y.T., Ghaedian R., Shetty K. (2005). Cranberry synergies for dietary management of *Helicobacter pylori* infections. Process Biochem..

[32] Apostolidis E., Kwon Y.I., Shetty K. (2008). Inhibition of *Listeria monocytogenes* by oregano, cranberry and sodium lactate combination in broth and cooked ground beef systems and likely mode of action through proline metabolism. Int. J. Food Microbiol..

[33] Lin Y.T., Labbe R.G., Shetty K. (2004). Inhibition of *Listeria monocytogenes* in fish and meat systems by use of oregano and cranberry phytochemical synergies. Appl. Environ. Microbiol..

[34] Wu VC H., Qiu X., Bushway A., Harper L. (2008). Antibacterial effects of American cranberry (*Vaccinium macrocarpon*) concentrate on foodborne pathogens. LWT—Food Sci. Technol..

[35] Aronsson K., Rönner U. (2001). Influence of pH, water activity and temperature on the inactivation of *Escherichia coli* and *Saccharomyces cerevisiae* by pulsed electric fields. Inonovative Food Sci. Emerg. Technol..

[36] Rahnayaka R.M.U.S.K. (2013). Antibacterial effect of malic acid against *Listeria monocytogenes*, *Salmonella enteritidis* and *Escherichia coli* in Mango, Pineapple and Papaya juices. Am. J. Food Technol..

[37] Xylia P., Chrysargyris A., Botsaris G., Tzortzakis N. (2021). Assessment of mint and pomegranate extracts/oils as antimicrobial agents to inhibit growth of *Escherichia coli* O157:H7 and *Listeria monocytogenes* on shredded carrots. Acta Hortic..

[38] Andrade M.A., Lima V., Sanches Silva A., Vilarinho F., Castilho M.C., Khwaldia K., Ramos F. (2019). Pomegranate and grape by-products and their active compounds: Are they a valuable source for food applications?. Trends Food Sci. Technol..

